# Dedicated comparatives aid comparisons of magnitude: a study with Pitjantjatjara-English bilinguals

**DOI:** 10.3389/fnhum.2024.1418797

**Published:** 2024-09-13

**Authors:** Luke Greenacre, Rebecca Defina, Skye Akbar, Jair E. Garcia

**Affiliations:** ^1^Monash Business School, Monash University, Melbourne, VIC, Australia; ^2^School of Languages and Linguistics, University of Melbourne, Melbourne, VIC, Australia; ^3^UniSA Business School, University of South Australia, Adelaide, SA, Australia; ^4^Physiology Department, Faculty of Medicine Nursing & Health Science, Monash University, Clayton, VIC, Australia

**Keywords:** language, comparisons, magnitude, number, quantity, extent

## Abstract

When expressing comparisons of magnitude, Pitjantjatjara, a language indigenous to the land now known as Australia, employs contextually driven comparators (e.g., Anyupa is tall. Uma is short) rather than a dedicated morphological or syntactic comparative construction (e.g., Anyupa is taller than Uma). Pitjantjatjara also has a small number of lexicalized numerals, employing ‘one’, ‘two’, ‘three’, then ‘many’. It is hypothesized that having dedicated comparatives in language and elaborated number systems aid comparisons of magnitudes. Fluent Pitjantjatjara-English bilinguals participated in tasks assessing their accuracy and reaction times when comparing two types of magnitude: numerosity (quantities of dots), and extent (line lengths). They repeated the comparisons in both languages on different days, allowing for the effect of language being spoken on responses to be assessed. No differences were found for numerosity; however, participants were less accurate when making comparisons of extent using Pitjantjatjara. Accuracy when using Pitjantjatjara decreased as the magnitude of the comparison increased and as differences between the comparators decreased. This result suggests a potential influence of linguistic comparison strategy on comparison behavior.

## 1 Introduction

Making comparisons is a fundamental component of decision making in everyday life. For instance, when shopping for groceries, one is faced with multiple complex tasks of comparison. Do you buy the bigger or smaller box of strawberries? The bigger box is more expensive, but is it cheaper per strawberry? What about if the smaller box is on sale? And what do you do if the strawberries in the smaller box look tastier? Each judgment requires comparisons of quantity and quality across multiple dimensions. This paper investigates possible linguistic and cultural differences in how people make these comparisons.

Our original motivation for this research arose from observations about differences in shopping behavior between people living in Pitjantjatjara- and English-speaking communities. During data collection for another study (Greenacre and Akbar, 2019), shopkeepers commented that some of the types of discounts they would commonly offer did not impact buyer behavior as they expected in Pitjantjatjara-speaking communities, i.e., offering $5 off an item did not seem to encourage people to buy it. There are several potential motivations for this reported difference in shopping behavior. We are mindful that, for some communities, stores are a newer concept, a direct result of colonization, which may impact how people navigate and interact with the retail environment. Differences in approach to discounts may also relate to various cultural differences, for example in how personal and cultural priorities are enacted. Finally, there is a possibility that it relates to linguistic differences, as there are differences both in how numbers are represented and in how comparisons are expressed between Pitjantjatjara and English. Most likely, any difference in shopping behavior is a combination of multiple factors. However, for this study, we focus on exploring the potential influence of linguistic differences through a set of experimental comparisons with Pitjantjatjara-English bilinguals in order to evaluate how likely it is for these factors to be playing a role.

Pitjantjatjara has been spoken by the peoples belonging to the deserts now also known as central Australia for many many thousands of years. It is spoken by approximately 3,500 people (Australian Bureau of Statistics, 2021), many of whom live in the Anangu Pitjantjatjara Yankunytjatjara (APY) lands located in the north-west corner of South Australia, with others living in communities across the Northern Territory, Western Australia, and South Australia. The disruption of colonization is very recent for many Pitjantjatjara speakers with people living today who were born into a traditional lifestyle and remember the move to European built settlements.

The Pitjantjatjara language is part of the Wati, or Western Desert, branch of the Pama-Nyungan language family, a group of closely related languages spoken across a large portion of central and western Australia. Pitjantjatjara is used as the dominant language of community life throughout the APY lands as well as in a few other remote communities. It is one of the few Australian Indigenous languages still being learnt as a first language by children (AIATSIS, 2020). Children growing up in APY communities typically learn Pitjantjatjara as their first language with English as a second language taught through the school system (Minutjukur et al., 2019; Defina, 2020). However, English is the dominant language of the school and services such as the health clinic in these communities. The crucial elements of Pitjantjatjara grammar for this paper relate to numbers and comparative construction, both of which are described below. For information about other aspects of the grammar, see Wilmoth (2023) and references therein.

Pitjantjatjara has a small set of lexicalized numerals: *kutju* ‘one’, *kutjara* ‘two’, and *mankur-pa* ‘three/a few’, where *-pa* is an epenthetic syllable added at the end of words which would otherwise end in a consonant (Goddard, 1985). Larger numerals can be expressed by additive combinations of these terms where the order of the numerals is variable and sequences of mankur can be reduplicated without the epenthetic *-pa*, e.g., *kutjara kutjara* ‘four’, *kutjara mankurpa* ‘five’, *mankur-mankurpa* ‘six’, *kutjara kutjara mankurpa* ‘seven’, *mankurpa mankurpa kutjara* ‘eight’, *mankurpa mankurpa mankurpa* ‘nine’, *kutjara kutjara mankur-mankurpa* ‘ten’, *kutjara mankur-mankur-mankurpa* ‘eleven’, *mankur-mankur-mankur-mankurpa* ‘twelve’. This strategy is rarely used in practice, however, with Pitjantjatjara speakers typically using the more the general *tjuta* ‘many’ which also functions as a general plural (more than two) marker, so that for instance *tjitji tjuta* means a set of children consisting of any number more than two, rather than strictly ‘many’ children. *Mankurpa* is also used to mean a ‘few’ rather than strictly ‘three’ (Goddard and Defina, 2020). Nowadays, Pitjantjatjara speakers also generally all speak English as well and use English numerals whenever they wish, including incorporating them into their Pitjantjatjara speech.

As part of this study, we asked 24 participants to say the number of items in a set (which ranged from 0 to 12): four participants used the combinations strategy (i.e., *mankurpa kutjara* ‘five’) all the way up to ‘twelve’; one switched to English after *mankurpa kutjara* ‘five’; seven switched to English numerals after *mankurpa* ‘three’; ten switched to English after *kutjara* ‘two’; and two participants used *tjuta* ‘many’ in reference to all numbers greater than ‘two’. Our impression from daily conversations in the community as recorded by Defina (2020) is that this task encouraged people to give very accurate responses (also see Discussion) and that in day-to-day conversation, people are more likely to either use the less precise *kutju*, *kutjara*, (*mankurpa*), *tjuta* system and not specify larger numbers, or just use English numerals. This can also be seen in the Pitjantjatjara translation of the New Testament (Tjukurpa Palya, 2019), another context which encourages more formal or exact language. There are 15 uses of *kutjara mankurpa* ‘five’ in the Pitjantjatjara version, compared to 45 uses of ‘five’ in the English reference version, 16 uses of *mankur-mankurpa* ‘six’, compared to 26 in the English, and no examples of any exactly named larger numerals, compared to 91 uses of ‘seven’ plus many other larger numbers in the English version.

Interestingly, in our numerosity naming task, people were more accurate using the combinations system all the way up to twelve (no errors) than when English numerals were used for numbers greater than five (8 errors, 3% error rate). This is likely because the design of the task encouraged an error where most numbers were presented in sequence, but one (eleven) was skipped over and the combinations strategy avoided this by grouping items into sets of two or three rather than following a particular sequence of numbers. This suggests there may be a difference in ordinality between the Pitjantjatjara combinations numerals and the English numerals, but this possibility is an opportunity for future research.

Much of the research on crosslinguistic variation in numerical cognition has focused on languages such as Pirahã and Mundurukú in Brazil (Gordon, 2004; Pica et al., 2004; Dehaene et al., 2008; Frank et al., 2008), and Warlpiri and Anindilyakwa in Australia (Harris, 1987; Butterworth et al., 2008) which have limited sets of lexicalized numerals. The hypothesis has generally been that people require cultural tools for working with numbers, specifically elaborated numeral lexicons, in order to accurately represent large numerosities across time, space, and modality. Before elaborating on these studies and how Pitjantjatjara compares, it is worth briefly reviewing the research on numerical cognition more generally.

Research on numerical cognition is extensive and has led to the proposal of various models including the Triple Code Model (TCM) (Dehaene, 1992) and A Theory of Magnitude (ATOM) (Walsh, 2003) among others (e.g., McCloskey et al., 1985; Feigenson et al., 2004; Leibovich et al., 2017; Sixtus et al., 2023). The current paper remains relatively agnostic between these various models and does not seek to discriminate between them. Rather, we base our investigation around common themes within this work. We do, however, follow Sixtus et al. (2023), Dos Santos (2022), and others in recognising the importance of discriminating four semantically distinct numerical concepts: ordinality, numerosity, number, and approximate magnitude.

Ordinality relates to relative order in a sequence, i.e., the knowledge that ‘seven’ is greater than ‘six’ because it comes later in the sequence of integers. It is often represented spatially and most crosslinguistic research relating to ordinality relates to differences in spatial mappings or representations of a mental number line (e.g., Dehaene et al., 1993; Zebian, 2005; Bender and Beller, 2011; Shaki and Gevers, 2011). The sense of ordinality and how it is represented appears to be strongly grounded in sensorimotor and cultural experiences, such as writing direction (Zebian, 2005) and cross-modal associations such as between space and time, i.e., is the future understood as being in front, behind, to the right, or the left (Fuhrman and Boroditsky, 2010). The concept of ordinality is required for lexicalized number sequences but extends beyond it to many other types of ordered sequences such as the alphabet, months of the year and seasons of the year (Casasanto and Pitt, 2019).

Numerosity relates to the quantity of elements within a set, for instance the number of people or chairs in a room. Number refers to a symbolic system of referring to numerosity, for instance the English numeral *twenty six* is a number. If you are in the room, you can determine if there are enough chairs by asking every person to sit down, one on each chair. If there are no people remaining without a chair and no extra chairs without a person, then the numerosity of people is the same as the numerosity of chairs. Numbers become particularly useful in situations where you cannot i.e. directly compare numerosities in such a way. Linguistic numerals are a primary, but not the only, form of numbers, for example tally marks and finger counting systems. Representations of numerosity are linked to our experiences of using number systems, e.g., through sensorimotor experiences of finger counting (Sixtus et al., 2023) as well as through the specificalities of our linguistic numeral systems. For instance, languages have different orderings of the decades and units in two-digit number words, such as twenty-four in French but four-twenty in German, and this has been shown to influence magnitude judgements when comparing two-digit numerals with speakers of languages like German more influenced by units than speakers of languages like French (Moeller et al., 2015; Van Rinsveld et al., 2016).

We all have the ability to easily and quickly recognise that a group of 50 people is larger than a group of 20. This ‘number sense’ relates to a basic skill in numerical cognition called the approximate magnitude system. Several theories and empirical evidence suggest that this system of approximating magnitudes is common across domains, including the estimation of size/length (the exterior dimensions of an object), time (duration of events), area (the surface of an object), quantity (how much of something appears) and density (how much of something appears in a fixed space) (e.g., Crollen et al., 2013; Walsh, 2003; Leibovich et al., 2017). Relevantly for this paper, studies have found that the estimation of numerosity and length have similar psychophysical profiles (Droit-Volet et al., 2008). This approximate magnitude sense is present from infancy in humans and has been found across several species (Feigenson et al., 2004; Agrillo et al., 2012). Both human and animal studies suggest the brain area responsible for this function is the right intraparietal sulcus (see Crollen et al., 2013).

Small numbers, typically those less than four, may additionally employ the parallel individuation system. There is some debate as to whether the parallel individuation system is employed separately or in conjunction with the approximate magnitude system (see Hyde, 2011 for a good summary). In either case, small numbers are processed by the brain near automatically with high levels of accuracy and confidence. In infancy, humans have been found to accurately recognise a specific magnitude, with the further ability to compare these magnitudes to determine which is larger developing quickly afterwards (Dehaene and Changeux, 1993).

Both systems for the perception of numerosity are potentially grounded in early sensorimotor experiences to some extent through common behaviors such as finger counting and spatial numerical associations (Sixtus et al., 2023). Both systems are not predicted to vary crosslinguistically and have been observed in studies with people speaking languages with limited number lexicons (e.g., Gordon, 2004). Such number sense is distinct from, yet underpins the concepts of ordinality and numerosity, both of which have been predicted to vary crosslinguistically.

One of the extremes in the linguistic variation of number representation is with languages such as Pirahã, Mundurukú, Warlpiri, Anindilyakwa, and Pitjantjatjara which have limited lexicalizations of number. Among these, Pirahã is the most extreme case known, with no lexicalized number terms at all, even for ‘one’. Researchers predicted that the lack of a lexicalized number system would impact how speakers of these languages represent numerosity. Studies with Pirahã speakers have indeed found that they cannot always accurately match exact numerosities, especially when the task requires memory to keep track of numerosity across time, space, or modality. While Gordon (2004) found a general decrease in accuracy with increasing numerosity, Frank et al. (2008) found that Pirahã speakers were generally accurate in tasks allowing direct matching, e.g., asking participants to place down a matching line of uninflated balloons for a provided line of spools of thread, but that their accuracy decreased when the potential for direct matching was decreased, either by placing the reference line of spools orthogonally to the line of balloons or by covering up the line of spools before participants placed their own line. Butterworth and colleagues (Butterworth et al., 2008) hypothesized that speakers of Warlpiri and Anindilyakwa, both Australian Indigenous languages with classifier systems including ‘one’, ‘two’, ‘many’, and ‘one’, ‘two’, ‘three’, ‘many’, respectively, would have similar difficulties with tasks requiring matching of numerosities across time, and modality. However, they found Warlpiri and Anindilyakwa speakers were just as accurate as English speakers, though they tended to reconstruct the spatial display suggesting they were relying on spatial strategies in place of numerals (Butterworth and Reeve, 2008).

Pitjantjatjara is a potentially interesting piece in this puzzle of whether and how linguistic differences in number influence numerosity. It is another language indigenous to Australia and shares some cultural similarities with other Australian Indigenous languages, particularly with Warlpiri. It is also a ‘one’, ‘two’, ‘three’, ‘many’ type language similar to Anindilyakwa, though Anindilyakwa arguably also has a low range base 5 system with number words for 5, 10, 15 and 20 (Stokes, 1982). If there are linguistic differences in the comparison of numerosities when speaking Pitjantjatjara and English it would suggest that the level of elaboration of our language’s numeral system, rather than just whether or not you have one (c.f. Pirahã) is important for facilitating the completion of tasks that require numerosity skills.

The other linguistic difference of interest between Pitjantjatjara and English is comparison. Pitjantjatjara primarily uses the juxtaposition strategy, rather than a dedicated morphological or syntactic comparative construction. To illustrate this approach, a person speaking Pitjantjatjara could say:

*1. Anyupa-nya   wara.   Uma-nya*   *mutumutu*.
     Anyupa-NOM^1^   tall.      Uma-NOM   short.
     ‘Anyupa is tall. Uma is short.’/ ‘*Anyupa is taller than Uma*.’

In this example, Anyupa is tall and Uma is short, and it is implied that ‘*Anyupa is taller than Uma’*. But to say Winmi*t*i is taller than Anyupa the Pitjantjatjara speaker would say:

2. *Winmi*t*i-nya*   *wara.   Anyupa-nya   mutumutu*.
     Winmi*t*i-NOM   tall.   Anyupa-NOM   short.
     ‘Winmi*t*i is tall.   Anyupa is short.’/ ‘Winmi*t*i is taller than Anyupa.’

Anyupa has not suddenly become short in the second example, it is just that within the set of Winmi*t*i and Anyupa she is the short one. The domain of comparison is determined by implicature and there can be alternative interpretations based on context. The sentence ‘*Anyupa is tall*’ could be used to imply that she is notably tall by conventional standards or that she is tall within the context of a specific set of people. In contrast, English comparatives, such as *‘Anyupa is taller than Uma’,* more explicitly specify the domain of comparison.

This type of comparative is called a conjoined comparative in linguistic typology (Stassen, 2013). Conjoined comparatives are utilized in several other languages indigenous to Australia, e.g., Warlpiri (Bowler, 2016), but not all, e.g., Wambaya employs a grammaticalized comparative (Nordlinger, 1998, p. 178). Kennedy (2009) predicts that such conjoined comparative constructions should not be acceptable in cases where there is very little difference between the things being compared (e.g., Winmi*t*i and Anyupa are almost the same height), also called crisp judgements, or when one descriptor is true of both (e.g., both Winmi*t*i and Anyupa are tall). However, Pitjantjatjara conjoined comparatives can be used in both these situations, and the same is noted for Warlpiri conjoined comparatives (Bowler, 2016).

Compared with numeral systems, there has been much less research on linguistic realizations of comparatives and their potential links to cultural and cognitive practices. However, based on a small typological survey, Dixon (2008) suggested that grammaticalized comparatives, like the English *-er*, are more likely to be found in societies with more complex features of material culture in particular those with more “stratified economic and political systems.” Conjoined comparatives, on the other hand, are more likely to be found in egalitarian societies (Dixon, 2008). This suggests a possible correlation between more explicit linguistic specification of domains of comparison and a cultural focus on comparison, e.g., through social stratification. Recent findings on the social impact of comparisons within social media are drawn to mind. It has been found that the increased use of social comparisons on social media can negatively impact well-being depending on individual circumstance (Meier and Johnson, 2022) and if linguistic, cultural, and cognitive differences in comparisons are found this would be a very interesting area for further applied research.

The idea that linguistic differences could relate to differences in cognition is known as linguistic relativity and has seen a huge amount of research since Whorf (1956). Wolff and Holmes (2011) provide an excellent overview of the field, in particular dividing linguistic relativity effects into seven distinct potential mechanisms. In our case, we are talking about what Wolff and Holmes (2011) refer to as ‘language as meddler’ and ‘language as augmenter’, the two subtypes of the ‘thinking with language’ category.

In the language as meddler subtype, people use language to solve a task which could be solved nonlinguistically, but when the linguistic and nonlinguistic codes are misaligned, this leads to interference resulting in slower or less accurate responses. For instance, English speakers are slower to discriminate light versus dark blue colors than Russian speakers who have an obligatory linguistic distinction between *siniy* “dark blue” and *goluboy* “light blue” (Winawer et al., 2007). This is the predicted mechanism for the potential influence of the comparison construction. One can discriminate approximate differences in the magnitude of numerosities and lengths without language, but people may nevertheless silently use language and for this task the standard Pitjantjatjara comparison construction may be less well aligned with the nonlinguistic task than the English construction. This is both because the Pitjantjatjara construction is less specific about the domain of comparison and because it leads to potential mismatches between linguistic descriptors and nonlinguistic attributes, e.g., Anyupa is described as short when both she and Winmi*t*i are tall. Thus, we predict that Pitjantjatjara speakers will be slower and or less accurate when comparing magnitudes than English speakers.

In the language as augmenter subtype, linguistic tools allow people to solve tasks they would not be able to solve without language. Number is a prime example of this subtype. Exact number words are a linguistic tool which people can use to solve tasks such as the accurate matching of numerosities when the reference is no longer available. Pirahã speakers did not have this tool and so were unable to accurately perform these tasks (Frank et al., 2008). For our study, the primary question is whether the linguistic tools provided by English numbers will be accessible to Pitjantjatjara-English bilinguals when they are speaking Pitjantjatjara, i.e., does this linguistic tool transfer (c.f. the Conceptual Transfer Hypothesis, Jarvis, 2007).

Linguistic relativity studies are often carried out between groups of monolingual participants in order to provide a clearer test of the potential difference between two linguistic strategies. However, more than half of the world’s population is bilingual (Grosjean, 2010) so any theories seeking to understand human cognition generally must account for bilingualism. There are also many situations where there are no, or very few, completely monolingual speakers of a language—this is the case with Pitjantjatjara where all adult speakers have experience with English through schooling, service encounters, and interactions with the wider community. Pitjantjatjara speakers also often speak other Indigenous languages and have done so long before English colonization. In such cases, it is not always necessary to establish monolingual baselines as it can be sufficient, and in fact potentially stronger as it eliminates other potential between-population effects, to show within-participant effects according to language context.

Bilingualism also poses interesting questions for linguistic relativity in how the use of multiple languages may or may not lead to different results. Generally, there are three potential outcomes that have been observed for linguistic relativity effects in bilinguals: (1) They behave like monolingual speakers of one of their languages [e.g., Yucatec Maya-Spanish bilingual children behave like Yucatec Maya monolinguals in spatial reference tasks regardless of the language of testing, though there is variation according to social variables (Chi Pech, 2024)]; (2) They behave like monolinguals of each language depending on the language the task is presented in [e.g., Italian-English bilinguals behave like monolinguals in tests of semantic effects of grammatical gender according to the language of testing (Kousta et al., 2008)]; (3) They do not behave like the monolingual speakers of either language, but instead somewhere in between [e.g., Dutch-French bilingual object categorization tends to converge in between Dutch and French monolingual categorization (Ameel et al., 2009)]. The outcome depends on their bilingual profile e.g., age of acquisition, language dominance, time spent in a community which uses the language, language of education etc., the specific task demands, and the mechanism of the linguistic relativity effect in question (Bassetti and Filipović, 2022).

For language as meddler relativity effects, bilinguals are likely to be influenced by the language of testing. This is because the effect is driven by the language the participant is actively drawing on during the task to solve the problem and these effects are known to disappear when participants are blocked from using language, e.g., with linguistic interference tasks (Winawer et al., 2007). The prediction is less clear for language as augmenter tasks—again the effect is driven by participants drawing on the linguistic structure during the task, but it is likely that bilinguals will draw on the structures which are most helpful for them in the task regardless of the language [e.g., Vietnamese-English bilinguals draw on lexicalized color concepts from both languages to solve color naming tasks (Jameson and Alvarado, 2003)]. However, it appears that some language tools do not always transfer between languages. For instance, Russian-English bilinguals learnt new arithmetic equations in one language and were then found to be more accurate for exact number information when tested in the language of training, there was no difference for approximate number or non-numerical information (Spelke and Tsivkin, 2001). Thus, it is not clear to what extent one would expect the influence of knowing an elaborated system of exact numerals in English would influence performance in exact numerosity tasks in Pitjantjatjara.

The present study seeks to investigate the hypothesis that the combination of the less elaborated number system in Pitjantjatjara and its use of conjoined comparatives will lead people to be slower and potentially less accurate in comparing magnitudes in Pitjantjatjara than in English. We used two comparison tasks, one exploring comparison of numerosities and another exploring comparison of extents. The combination of these tasks allows us to evaluate the hypothesis across domains. Experiments were carried out with a group of people fluent in both Pitjantjatjara and English. Participants performed each task twice, once in each language, so that we could carry out a within-participant language comparison.

## 2 Methods

We tested 16 people with each person first completing the experiments in one language (Pitjantjatjara or English) and then repeating the experiments in the other language at least one day later. The language of the experiment was established by conversation with the experimenter prior to the tasks, as well as through all instructions and questions within the experiment. Choices (and their accuracy) and reaction times were recorded with a response box in Direct RT. At the end of the comparison tasks, participants were asked to verbally respond to demographic questions and to name the quantities of several clouds of dots. Their language use in the final verbal task was used as an indicator of whether they were suitably primed in the experiment language or not.

All methods and instructions were co-designed and translated with community members to ensure understanding and cultural appropriateness, following best practice for psycholinguistic research in understudied communities outside of lab situations (e.g., Speed et al., 2017; see also Akbar et al., 2023 for discussion of working in mixed Indigenous and non-Indigenous research teams, with specific mention of this project). The research team was already familiar with the community and the language which aided in developing the initial version of the instructions with subsequent testing and revision undertaken over multiple iterations. Design choices involved selecting how we referred to the dots and lines, how we phrased the comparison questions, how large the numerosities we tested were and how many trials we included. The largest change within this piloting process was from presenting the comparators sequentially and thus relying on a memory component for the comparison, to presenting the comparators simultaneously on a single screen to allow for more direct comparison. While previous studies (e.g., Frank et al., 2008) have found that language effects on numerosity comparisons are particularly apparent when the task requires memory rather than direct comparison, piloting showed that a sequential comparison task generated confusion, with participants indicating that they did not feel confident in what they were comparing. In retrospect, this relates to our comparison hypothesis, see Discussion. While a memory-based comparison of numerosities would be preferable based on the prior literature, it remains a question for future research. Nevertheless, given that Frank et al. (2008) also found an effect when the reference line was provided orthogonally rather than parallel to facilitate direct matching, it is feasible that an effect can still be found with dot clouds.

### 2.1 Participants

Sixteen participants were recruited at a community center in Pukatja, within the APY Lands in South Australia. All participants self-reported fluency in both Pitjantjatjara and English and were employed in roles where they were regularly required to draw on both languages, such as Pitjantjatjara-English interpreters and translators or those working closely with English monolinguals. All participants comfortably interacted with the research team in both languages throughout the course of the study. The majority of participants (15/16) reported learning Pitjantjatjara as their first language, with the remaining person reporting they learned English first. For all participants, their second language (English or Pitjantjatjara) was acquired during childhood. All participants reported currently using Pitjantjatjara as their primary home language. Some participants additionally reported fluency in Arrernte (1), Ngaanyatjarra (1), Yankunytjatjara (2), or Yolngu (1).

Of the 16 participants, 15 indicated their sex was female (94%) with an average age of 40.9 years (SD = 11.7). All 16 participants reported normally using their right hand to write, and 15 reported using their right hand to throw a ball. Participants were asked if they needed reading glasses or contact lenses, with all those reporting such a need (*n* = 3) wearing them.

An additional 10 participants were excluded from the final sample. These people either did not return to complete the experiments in the second language (*n* = 5) or during the subsequent spoken language task, where they were asked to say aloud numbers, they did not respond in the target experiment language (*n* = 5), suggesting the language prime may not have been maintained for them.

### 2.2 Materials and procedure

#### 2.2.1 Dot quantity (numerosity) experiment

**Figure 1:**
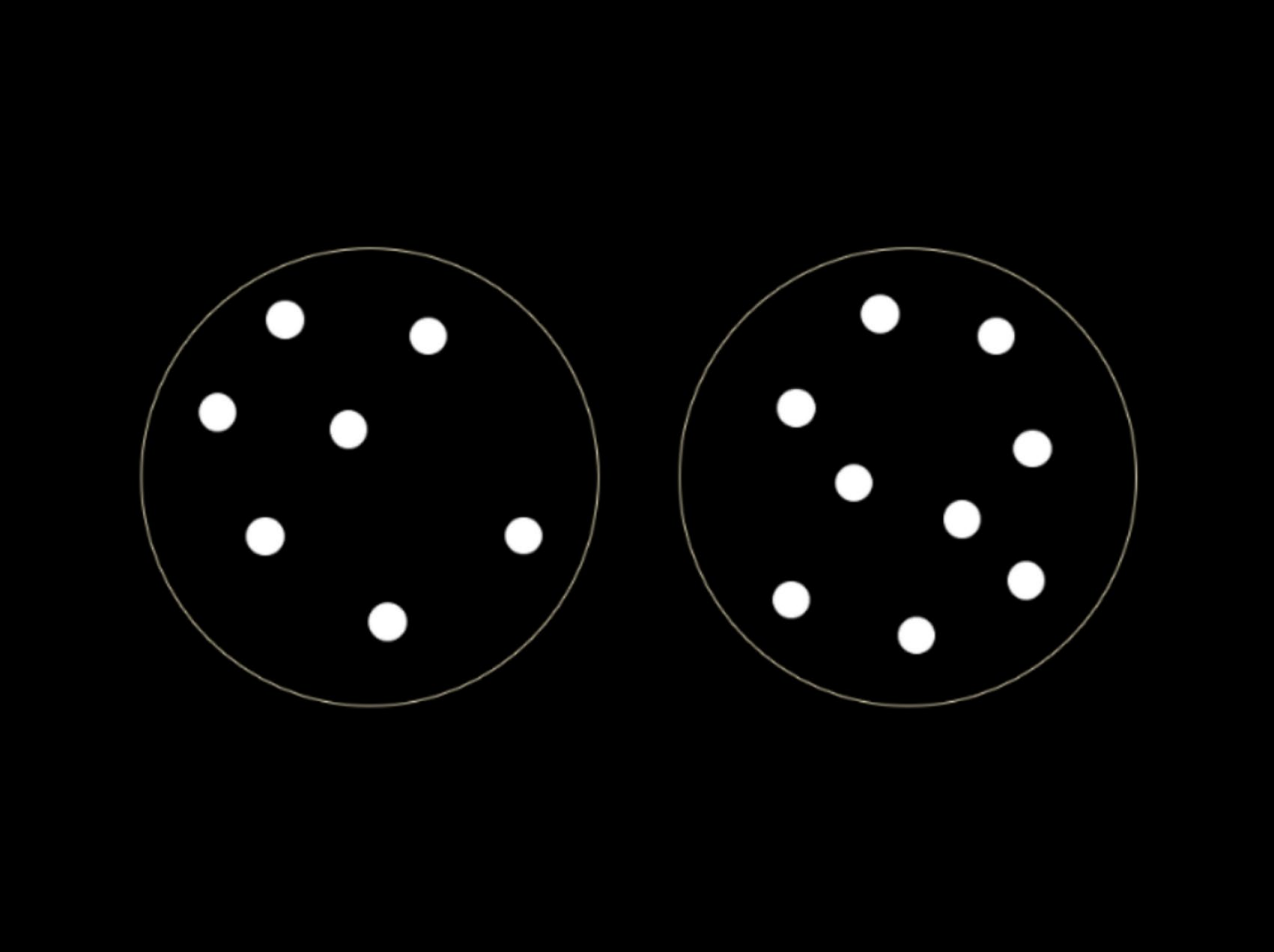
Example trial in the dot quantity experiment, here the right cloud has more dots.

This experiment consisted of ten trials. Each trial consisted of a quantity comparison between two clouds of dots simultaneously visible on the screen with participants prompted to identify which had more dots (e.g., Figure 1). Five pairs of quantities were compared (2, 3), (5, 6), (8, 9), (2, 4) and (7, 9), with each pair shown twice—once with the smallest on the left and again with the largest on the left. These numbers were chosen as they include pairs below the subitizing range, above the subitizing range, across the subitizing range boundary, and combinations of odd and even numbers. The subitizing range includes numbers whose magnitude can be assessed highly rapidly and accurately, typically the numbers 1 through 4. The order of presentation of the pairs was randomized between participants using the Direct RT software. Dots were white on a black background.

#### 2.2.2 Line length (extent) experiment

**Figure 2:**
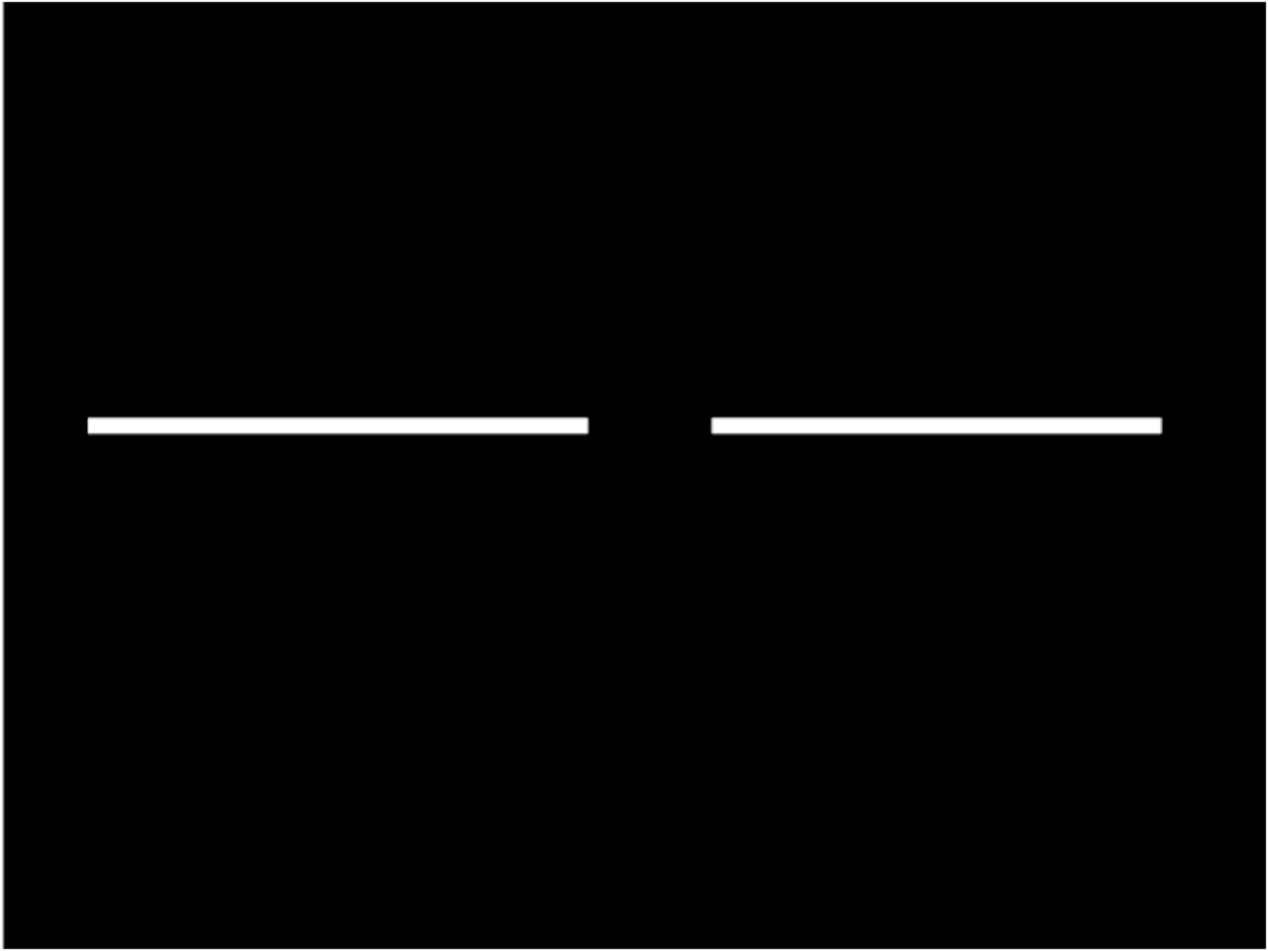
Example trial in the line length (extent) experiment, here the left line is longer.

This experiment also consisted of ten trials. Each trial consisted of an extent comparison of two lines shown simultaneously horizontally next to each other (e.g., Figure 2). Five pairs of lines were compared (25 mm, 30 mm; which is a ratio of 5:6), (45 mm, 50 mm; ratio 9:10), (30 mm, 50 mm; ratio 3:5), (25 mm, 50 mm; ratio 1:2) and (25 mm, 60 mm; ratio 5:12), with each pair shown in both orders. The absolute lengths were chosen based on visual space available on the monitor. The pairs were chosen as they compare sets of similar length lines (ratios 5:6 and 9:10), and dissimilar lines where one is just below (5:12), exactly (1:2), and just above (3:5) double the other. Lines were white on a black background.

The two experiments were programmed in Direct RT and displayed on a 13 inch laptop display. Participants were first given instructions for using the response box and completed a sample task for the dot quantity experiment. They then completed the dot quantity experiment, where they were asked to indicate which cloud had the most dots. Directly after the dot quantity experiment, they received instructions for the line length comparison experiment and completed a sample task. They then completed the full line length experiment, indicating for each trial which line was longer. In all comparison tasks, participants were instructed to answer as quickly as they could. If they took longer than 1,500 ms to respond they were asked to ‘press the button faster/button warpungkula puuntanama’. Between all trials a centrally located + was shown on the screen for 500 ms.

After the two experiments, participants were prompted, under no time limit and within DirectRT, to undertake a separate verbal task in which they named how many dots were present in a sequence of sequential dot clouds. The quantities shown in sequence were 1, 2, 3, 4, 5, 6, 7, 8, 9, 10, 12 and zero. This naming task was included to confirm that the appropriate language had been effectively primed, with the expectation that participants would use the same language as that used in the experiment if suitably primed in that language. Participants also verbally answered demographic questions relating to age, sex, handedness, and language background. The experimenter typed their answers into the computer and triggered the next question. The participants returned the next day (or in subsequent days) to complete the experiment in the other language, with the order of languages chosen on a rotating basis between participants. The demographic questions were only answered in the first session.

All instructions in the experiments were provided in both written and spoken format. A professional Pitjantjatjara/English translator recorded all the audio of the spoken instructions in both languages, and this was automatically played during the experiment. The speaker is a Pitjantjatjara as first-language speaker and member of the wider Pitjantjatjara community, though does not live in the community where participant recruitment took place. The instructions were specifically written to be clear in both languages and used common terminology. All translations were first written in English by the researchers and then forward and back translated across several iterations with different translators to ensure accuracy of translations. Where terminology was found to be difficult to translate substitute terminology was used until we had clear and equivalent instructions in both languages.

In the dot quantity experiment the circles containing the dots were called baskets/piti and the dots were stones/puli. The instructions, which were provided across several slides in the experiment, are provided below. The first line is the Pitjantjatjara instruction as provided (without the morpheme break hyphens), and the English translation is the English instruction as provided. The participants completed a sample task with feedback on their speed and accuracy, and then moved onto the main experimental tasks.

3. *Nyangatja   puli*
      this.one      stone
      ‘This is a stone’
      (with a white dot shown)
4. *Nyangatja   piti   kutjara*
     this.one      bowl      two
     ‘These are two baskets’
     (with two white circles shown)
5. *Kutjupa-ngu   puli   tjuta   tjunu   piti-ngka   un-ngu*
     another-ERG   stone   PL   inside   bowl-LOC   distribute-PST
     ‘Someone put stones in the baskets’
     (with quantities of dots now shown in the circles)
6. *Yaaltji-ngka   puli   tjuta   ngari-nyi?*
     where-LOC    stone    PL   lie-PRS
     ‘Which basket has the most stones?’
     (same circles and dots as on prior screen)
7. *Button    puunta-ra    panya    puli    tjuta    mulapa    tjara*
      button    press-IMP    ANAPH    stone    PL    true    PROP
      ‘Press the button on the same side as the basket with the most stones’
      (response box shown on screen with arrows to the left- and right-side buttons)

In the line length experiment the lines were described as sticks/punu. The instructions provided are listed below. As with the dot quantity experiment, participants then completed a sample task with feedback on their speed and accuracy, and then moved onto the main experimental tasks.

8. *Nyangatja    punu*
      this.one      stick
      ‘This is a stick’
      (with a solid white line shown on screen)
9. *Ka      nyangatja      punu      kutjara*
       and.DS      this.one      stick      two
       ‘You will see two sticks’
       (with two white lines shown on screen)
10. *Nyuntu      nyaku-la      tjakultju-nama      yaaltji      punu      wara?*
       2.SG.ERG      see-MV      report-CONT.IMP    where      stick      long
       ‘You say which stick is longer’
       (with the same two lines as on prior screen)
11. *Kampa      yaaltji      punu      wara?      Nyaku-la-mpa      button      puunta-ra*
       side           where      stick      long      see-MV-DIS      button      press-IMP
       ‘Press the button on the same side as the longer stick’
       (response box shown on screen with arrows to the left- and right-side buttons)

### 2.3 Models

Four mixed linear models were fitted for each of the following four dependent variables: Dot Quantity (Numerosity)—Reaction time (measured in milliseconds); Dot Quantity (Numerosity)—Choice accuracy (correct/incorrect); Line Length (Extent)—Reaction time (milliseconds); Line Length (Extent)—Choice accuracy (correct/incorrect). Prior to analysis all observations with reaction times exceeding 3,000 ms were excluded from analysis, including the corresponding choice observation. Mixed models included fixed parameters for the main effect of language, the magnitude (quantity or length) of the lower value in the pair in the trial, the absolute difference (in quantity or length) between the pairs in the trial, and the interactions between language and the latter two. A random intercept term consisting of the ID number for each participant was included to account for repeated observations recorded from each participant. In the case that the full model did not converge, two reduced models were estimated, each including a single interaction term to test for their significance individually.

The choice models assumed a Binomial distribution for the binary (correct, incorrect) response and were fitted with the glmer routine available in the library lme4 v 1.1-23 (Bates et al., 2015) for R v 4.0.2 (R Core Team, 2020). Reaction time was log-transformed to ensure the normality of the residuals in those models with them fitted with the lmer routine available in the same package. The significance of the predictor variables was tested using an Analysis of Deviance with Type III Wald chi-square tests using the car package (Fox and Weisberg, 2019). Confidence intervals for the means predicted by the model were estimated by bootstrap simulation with 10,000 iterations using the merTools library (Knowles and Frederick, 2020).

## 3 Results

Our hypothesis predicted participants would be less accurate and/or take longer to process comparisons in Pitjantjatjara. This was tested across two domains, quantity (numerosity) and length (extent). Results in fact only showed a language difference in comparisons of length (extent).

For the dot quantity (numerosity) experiment, prior to outlier removal, the 16 participants were 92.8% accurate across both languages and were generally slow in responding compared to what is typically observed in estimation tasks, on average taking 3,339 ms. After outlier removal, (96 observations removed) accuracy was 96.0% and average response time was 1,408 ms. This result suggests participants prioritized accuracy and compared the exact quantity of dots rather than estimating. Informal debriefing with participants confirmed this observation, participants felt highly motivated to do the experiment ‘well’ and hence they took time to count the dots as accurately as they could rather than estimating the quantity as was instructed in the experiment. The full model did not converge for the *choice accuracy* model, so the reduced models were estimated. Only the model including the language * lowest number interaction term converged, see Table 1. No significant differences based on language used for the experiment were identified. For the reaction time data, the full model with both language * lowest number and language * absolute difference interaction terms converged, see Table 2. Again, no significant differences based on language were identified. The only significant effect was the lowest number. Participants tended to respond faster when the quantity of the smallest comparator was less.

**Table 1:** Model results for choice accuracy for the dot quantity (numerosity) experiment.

Model term	Chi square	df	*p*-value
Intercept	3.60	1	0.058
Language	1.12	1	0.291
Lowest number	0.557	1	0.456
Absolute difference	0.684	1	0.408
Language * Lowest number	0.556	1	0.456

**Table 2:** Model results for reaction time for the dot quantity (numerosity) experiment.

Model term	Chi square	df	*p*-value
Intercept	1987	1	<0.001***
Language	0.002	1	0.962
Lowest number	21.1	1	<0.001***
Absolute difference	0.392	1	0.531
Language * Lowest number	0.023	1	0.879
Language * Absolute difference	0.017	1	0.898

***p ≤ 0.001.

In contrast, the 16 participants did appear to approximate for the line length (extent) task. Prior to outlier removal, accuracy was 90.3% with an average response time of 1,307 ms. After outlier removal, (17 observations) accuracy was 90.8% with an average response time of 1,066 ms. While accuracy was broadly similar between the two experiments, responses were faster compared to the dot quantity (numerosity) experiment. Reaction time was only influenced by the absolute difference between the numbers in the pairs (chi-squared = 19.1, *p* < 0.001) with participants responding to line pairs that had a greater difference between them faster than more similar lines, see Table 3. There were no significant differences based on language.

**Table 3:** Model results for reaction time for the line length (extent) experiment.

Model term	Chi square	df	*p*-value
Intercept	1,033	1	<0.001***
Language	0.189	1	0.664
Lowest number	0.003	1	0.954
Absolute difference	19.1	1	<0.001***
Language * Lowest number	0.045	1	0.831
Language * Absolute difference	0.422	1	0.516

***p ≤ 0.001.

**Figure 3:**
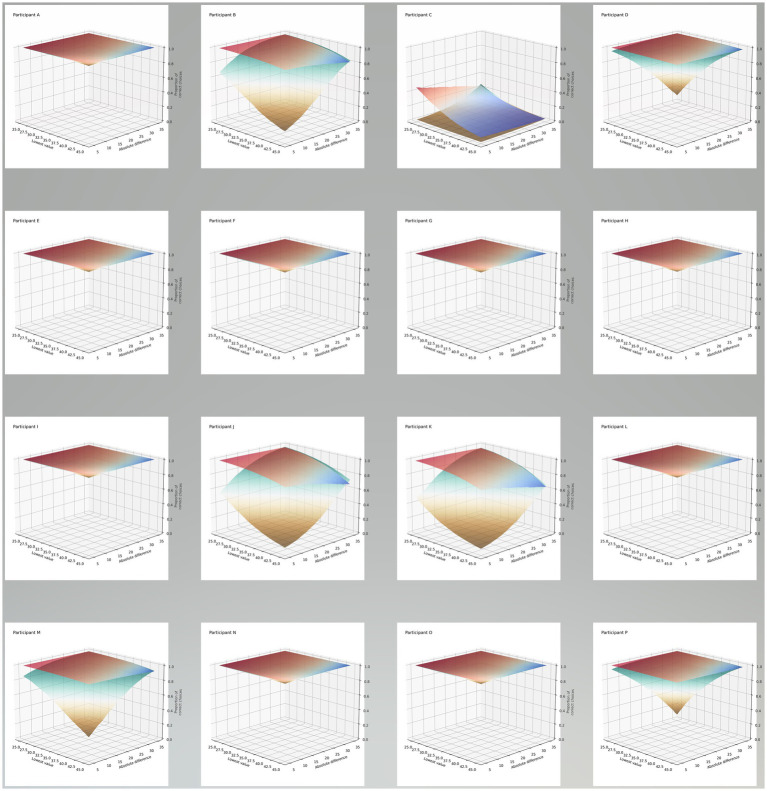
Surface plots summarizing the model. Each surface represents the proportion of correct choices (z-axis) for the various lower values in each pair of lines shown to participants and for the absolute differences between each of those pairs, across the two languages: English (red-blue surface) and Pitjantjatjara (brown-green surface).

The full model for choice accuracy did not converge for the line length (extent) experiment. Only the model including the language * absolute difference interaction term converged, see Table 4. There was a significant difference between the languages in responses for the absolute difference between the pairs of lines (chi-squared = 4.50, *p* = 0.034). When we plot the data for each participant (Figure 3) we can see several consistent patterns. Take participant B as an exemplar, when using English there is a higher overall proportion of correct choices compared to Pitjantjatjara, with this high proportion decreasing slightly as the lowest value in the pair increases. For Pitjantjatjara there is a stronger effect with proportion of correct choices decreasing as both the lowest value in the pair increases and the difference between the pairs decreases.

**Table 4:** Model results for choice accuracy for the line length (extent) experiment.

Model term	Chi square	df	*p*-value
Intercept	6.40	1	0.011*
Language	9.92	1	0.002**
Lowest number	6.11	1	0.013*
Absolute difference	0.427	1	0.514
Language * Absolute difference	4.50	1	0.034*

*p ≤ 0.05, **p ≤ 0.01.

## 4 Discussion and conclusion

We sought to investigate the hypothesis that the limited lexicalization of number and the use of conjoined comparatives in Pitjantjatjara will lead people to be slower and potentially less accurate in comparing magnitudes in Pitjantjatjara than in English. We found evidence to support the hypothesis, but only in one domain. Participants were less accurate when making comparisons of extent using Pitjantjatjara, with accuracy decreasing with the general difficulty of the comparison, namely as magnitudes increased and the crispness of the comparison increased, i.e., with decreasing difference between comparators.

Notable in the findings was the difficulty in getting participants to use approximation when doing the dot quantity (numerosity) experiment. Participants wanted to do the experiment ‘well’ and thus assessed exact quantities via counting to maximize accuracy based on the reaction times observed and debriefing. This focus on performing the task accurately matches recent findings in how cultures indigenous to the land now known as Australia approach cognitive assessment tasks (e.g., Dingwall et al., 2017). It also aligns with Hofstede and colleagues’ findings that Indigenous people tend to have a significantly higher tendency to avoid uncertainty than non-Indigenous Australians (Hofstede et al., 2005). This focus on accuracy likely appeared more in the quantity than the extent task because counting provided a strategy for accuracy in the numerosity task while there was no such readily available strategy for the extent task.

Thus, our two tasks also likely differ in the relevant linguistic relativity mechanism: the numerosity task would now be a ‘language as augmenter’ task as accurate discrimination of the dot clouds was not possible without a tool such as lexical numerals; while the extent task remains a ‘language as meddler’ task as estimation can be done without linguistic tools.

The equally high accuracy in the numerosity task when completed in both English and Pitjantjatjara would then suggest that participants were able to draw on the tool of lexicalized numerals as desired regardless of the language context. This would fit with other research in bilingual cognition where concepts are known to transfer between languages (e.g., Jarvis, 2007) and people are seen to draw on linguistic tools from all of their languages as needed to solve tasks (e.g., Jameson and Alvarado, 2003). This could suggest that language as augmenter effects may not show for bilinguals when tested within-participant based on language context.

Another potential explanation for the non-significance of the language effect in the numerosity task is that it was not mediated through memory. Prior research (e.g., Frank et al., 2008) has shown that influences of lexicalized number systems on estimations of numerosity are observed primarily when the task involves memory, or tracking numerosity across time, space, or modality. The lack of a memory component may have allowed participants to solve the task by directly matching the dots from one cloud to the other. This would have allowed them to accurately solve the task nonlinguistically. We believe this is less likely as the dots were randomly placed within the dot clouds and Frank et al. (2008) found that direct matching was only used when the sets were aligned in order to facilitate matching, also because participants reported using counting as a strategy. However, without a memory mediated task we cannot rule out the possibility of direct matching. Our pre-testing showed the need for a simultaneous comparison task on a single screen to avoid confusion with our participant group. While the avoidance of a memory-based comparison introduced this potential confound, this pre-testing finding is in fact in line with the comparison construction hypothesis which suggests that the conjoined comparison construction leads to greater ambiguity in the domain of comparison. Further research examining both simultaneous and sequential comparison tasks together may offer greater insights on the interactions between memory and the potential influences of both number lexicalization and comparison grammaticalization.

We did find a difference between Pitjantjatjara and English in the extent comparison task. This suggests that the use of conjoined comparatives, rather than a dedicated morphological or syntactic comparative construction, may have a ‘language as meddler’ effect leading people to have a slower processing or more varied, and so less-accurate, judgment of comparison. This could be due to a less specified domain of comparison in conjoined comparatives than what is expressed with dedicated morphological or syntactic comparative constructions. This is a particularly unique finding in the literature.

An avenue for further research to explore both the number lexicalization and comparison grammaticalization effects is to investigate across multiple languages. It is estimated that there are 123 Indigenous languages or language varieties currently spoken across Australia (AIATSIS, 2020) but there are still few studies looking at multiple languages simultaneously, with them typically studied in isolation. Likewise, around the world, languages with limited lexicalizations of number (i.e., where some integer numbers are not named) and/or use of conjoined comparison tend to be smaller, lesser-studied languages and are often investigated in isolation. Identifying language combinations with similar comparative constructions but differences in the elaboration of the number system and vice versa similar lexicalizations of number but differences in comparative constructions will allow for these two potential influences to be isolated and their individual effects on magnitude perceptions to be considered.

Overall, we find evidence that the use of dedicated grammaticalized comparison constructions impacts magnitude perceptions with regard to extent. We find this by examining the case of Pitjantjatjara- and English-speaking bilinguals. The research was originally motivated by observing people living in Pitjantjatjara- and English-speaking communities while they were shopping and hearing from retailers that they identified that some common discount strategies did not always function as expected (Greenacre and Akbar, 2019). It is hoped that exploring the impact of language on magnitude perceptions will allow for the development of services, such as stores, that better meet the needs of those speaking a diverse set of languages.

## Data Availability

The original contributions presented in the study are included in the article/Supplementary material, further inquiries can be directed to the corresponding author.

## Footnotes

^1^Abbreviations used: 2 ‘second person’, CONT ‘continuative’, DIS ‘discourse marker’, DS ‘different subject’, ERG ‘ergative’, IMP ‘imperative’, LOC ‘locative’, MV ‘medial verb’, NOM ‘nominative’, PL ‘plural’, PRS ‘present’, PROP ‘proprietive’, PST ‘past’, SG ‘singular’.
